# Fabrication of a novel magnetic topological heterostructure and temperature evolution of its massive Dirac cone

**DOI:** 10.1038/s41467-020-18645-9

**Published:** 2020-09-24

**Authors:** T. Hirahara, M. M. Otrokov, T. T. Sasaki, K. Sumida, Y. Tomohiro, S. Kusaka, Y. Okuyama, S. Ichinokura, M. Kobayashi, Y. Takeda, K. Amemiya, T. Shirasawa, S. Ideta, K. Miyamoto, K. Tanaka, S. Kuroda, T. Okuda, K. Hono, S. V. Eremeev, E. V. Chulkov

**Affiliations:** 1 Department of Physics, Tokyo Institute of Technology, Tokyo, 152-8551 Japan; 2 Centro de Física de Materiales, CFM-MPC, Centro Mixto CSIC-UPV/EHU, Apdo.1072, 20080 San Sebastián/Donostia, Basque Country Spain; 3 IKERBASQUE, Basque Foundation for Science, 48011 Bilbao, Spain; 4 Research Center for Magnetic and Spintronic Materials, National Institute for Materials Science, Tsukuba, 305-0047 Japan; 5 Graduate School of Science, Hiroshima University, Higashi-Hiroshima, 739-0046 Japan; 6 Materials Sciences Research Center, Japan Atomic Energy Agency, Sayo, Hyogo 679-5148 Japan; 7 Institute of Materials Science, University of Tsukuba, 1-1-1 Tennoudai, Tsukuba, 305-8573 Japan; 8 Center for Spintronics Research Center, University of Tokyo, Tokyo, 113-8565 Japan; 9 Institute of Materials Structure Science, High Energy Accelerator Research Organization, Tsukuba, Ibaraki 805-0801 Japan; 10 Department of Materials Structure Science, School of High Energy Accelerator Science, The Graduate University for Advanced Studies (SOKENDAI), Tsukuba, Ibaraki 305-0801 Japan; 11 National Institute of Advanced Industrial Science and Technology, Ibaraki, 305-8560 Japan; 12 UVSOR Facility, Institute for Molecular Science, Okazaki, 444-8585 Japan; 13 Hiroshima Synchrotron Radiation Center, Hiroshima University, Higashi-Hiroshima, 739-0046 Japan; 14 Institute of Strength Physics and Materials Science, Tomsk, 634055 Russia; 15 Tomsk State University, Tomsk, 634050 Russia; 16 Saint Petersburg State University, Saint Petersburg, 198504 Russia; 17 Donostia International Physics Center (DIPC), Paseo de Manuel Lardizabal, 4, 20018 San Sebastián/Donostia, Basque Country Spain; 18 Departamento de Física de Materiales, Facultad de Ciencias Químicas, UPV/EHU, Apdo. 1072, 20080 San Sebastián, Basque Country Spain

**Keywords:** Ferromagnetism, Surfaces, interfaces and thin films, Topological insulators

## Abstract

Materials that possess nontrivial topology and magnetism is known to exhibit exotic quantum phenomena such as the quantum anomalous Hall effect. Here, we fabricate a novel magnetic topological heterostructure Mn_4_Bi_2_Te_7_/Bi_2_Te_3_ where multiple magnetic layers are inserted into the topmost quintuple layer of the original topological insulator Bi_2_Te_3_. A massive Dirac cone (DC) with a gap of 40–75 meV at 16 K is observed. By tracing the temperature evolution, this gap is shown to gradually decrease with increasing temperature and a blunt transition from a massive to a massless DC occurs around 200–250 K. Structural analysis shows that the samples also contain MnBi_2_Te_4_/Bi_2_Te_3_. Magnetic measurements show that there are two distinct Mn components in the system that corresponds to the two heterostructures; MnBi_2_Te_4_/Bi_2_Te_3_ is paramagnetic at 6 K while Mn_4_Bi_2_Te_7_/Bi_2_Te_3_ is ferromagnetic with a negative hysteresis (critical temperature  ~20 K). This novel heterostructure is potentially important for future device applications.

## Abstract (web summary)

Magnetic topological heterostructures are promising devices to manipulate emergent quantum effects. Here, Hirahara et al. fabricate a novel magnetic topological heterostructure with a massive Dirac cone which becomes a massless one tuned by temperature.

## Introduction

Inducing magnetism in topological insulators (TI) leads to exotic quantum phenomena^1^ such as the quantized anomalous Hall effect^2,3^, topological magnetoelectric effect^4^, and the realization of chiral Majorana Fermions^5^. In terms of the electronic structure, the surface Dirac cone (DC) of TI will be gapped due to the time-reversal symmetry breaking. So far most efforts to incorporate magnetism into TI have been performed by doping magnetic impurities inside the TI crystals. However, this approach induces inhomogeneity to the samples and the formation of impurity states. This has led to the unfortunate situation that the experimental observation of the quantum effects is limited to very low temperatures^6^. Furthermore, it has been reported that a DC gap can arise due to nonmagnetic origins^7^.

Recently, a novel method to combine magnetism with TI has been proposed, which is called magnetic extension^8,9^. The idea is to place a magnetic monolayer inside the topmost quintuple layer (QL) of the TI. This was experimentally realized by self-organized incorporation of a Mn monolayer inside Bi_2_Se_3_, resulting in the formation of a MnBi_2_Se_4_/Bi_2_Se_3_ heterostructure^10^. This method was further extended and complicated heterostructures composed of MnBi_2_Se_4_ and Bi_2_Se_3_, or MnBi_2_Te_4_ and Bi_2_Te_3_, were experimentally fabricated^11,12^. Furthermore, MnBi_2_Te_4_ and MnBi_4_Te_7_ called “intrinsic magnetic TIs” were realized as bulk single crystals^13–23^.

Concerning the surface DC of these systems, there is a debate about the presence/absence of the gap itself as well as its relation with the magnetic properties of the system. It has been shown that the DC gap is observed below the Curie temperature (*T*_C_) but closes above it, indicating that the magnetic order and DC gap are correlated with each other^12^. On the other hand, there is a work reporting that the DC gap persists above the Néel temperature (*T*_N_) up to 300 K and this was explained by the formation of an instantaneous out-of-plane field generated by strongly anisotropic spin fluctuations^13^. Similar explanation was employed for the presence of the massive DC above *T*_N_ in refs. ^16,22^. On the other hand, it was claimed that the DC is massless even at temperatures lower than *T*_N_^17–21^. The origin was described as a result of the neutralization of the net magnetic moments due to the presence of different magnetic domains within the sample^17,19–21^, or the weak hybridization between the local moments and the Dirac electrons^18^. There is another work that insists that the DC was massless at 25 K, which is above *T*_N_, but no comment was made on the behavior below *T*_N_ in ref. ^15^.

As such, the presence of a DC gap as well as its closing in a magnetic TI is still not fully understood even in stoichiometric well-ordered samples. Furthermore, to the best of our knowledge, there has been no experimental work showing the systematic change in the gap size deduced from sharp spectral features over a wide range of temperature. One of the reasons may be the two-dimensional character of the magnetic Mn layers with the distance between the adjacent layers of at least  ~1 nm. Since the interlayer magnetic interaction is expected to be weak and prone to fluctuations, the magnetization and hence the DC gap may be strongly influenced by unintended defects that are inevitably present in the fabricated crystals. Thus a system with a shorter distance and a stronger magnetic interaction between the stacked layers is desirable to further understand the relationship between the DC gap and the magnetization of the system. In this respect, ref. ^24^ has predicted an interesting heterostructure where multilayers of MnSe or MnTe are embedded in the topmost layers of Bi_2_Se_3_ or Bi_2_Te_3_. Although the Mn layers may couple antiferromagnetically, the stronger interlayer magnetic interaction should lead to a situation where the correspondence between the magnetic properties and the DC gap should be clear-cut compared to the previously studied systems.

In the present work, we report on the successful experimental fabrication of such an exotic magnetic topological heterostructure. By co-depositing Mn and Te on Bi_2_Te_3_, we observed a massive DC with a gap of 40–75 meV at 16 K. There were two photoemission intensity peaks that could be unambiguously identified as the upper and lower DC and its temperature evolution was traced. We found that the gap size decreases by raising the temperature and eventually a blunt transition to a massless DC occurrs at 200–250 K. The atomic structure was verified as a mixture of MnBi_2_Te_4_/Bi_2_Te_3_ and Mn_4_Bi_2_Te_7_/Bi_2_Te_3_ heterostructures and the observed massive DC was ascribed to the latter. The system showed long-range magnetic order with a critical temperature (*T*_c_) of  ~20 K and showed a negative hysteresis likely due to the antiferromagnetic coupling between the Mn magnetic moments of the two heterostructures. Thus, our results show for the first time that the Dirac point gap of the interface topological state of the magnetic topological heterostructure eventually closes, albeit at a temperature well above the magnetic *T*_c_.

## Results

### The surface Dirac cone and its temperature evolution

**Fig. 1:**
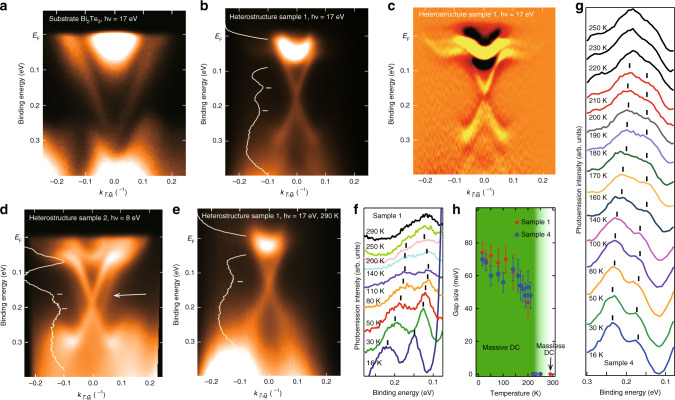
Dirac-cone dispersion and its temperature evolution. **a** Band dispersion of the substrate Bi_2_Te_3_ film measured along the $\bar{\Gamma }-\bar{{\rm{M}}}$ direction taken at *hν* = 17 eV at 16 K. **b** Band dispersion of the heterostructure for sample 1 measured along the $\bar{\Gamma }-\bar{{\rm{M}}}$ direction taken at *hν* = 17 eV at 16 K. The white line shows the energy distribution curve (EDC) at the $\bar{\Gamma }$ point. **c** The second derivative with respect to the energy of the band dispersion image in (**b**). **d** Band dispersion of the heterostructure for sample 2 measured along the $\bar{\Gamma }-\bar{{\rm{M}}}$ direction taken at *hν* = 8 eV at 30 K. The white line shows the EDC at the $\bar{\Gamma }$ point. The arrow shows additional features that likely corresponds to the DC of MnBi_2_Te_4_/Bi_2_Te_3_. **e** Band dispersion of the heterostructure for sample 1 measured along the $\bar{\Gamma }-\bar{{\rm{M}}}$ direction taken at *hν* = 17 eV at room temperature. The white line shows the EDC at the $\bar{\Gamma }$ point. **f**, **g** Temperature dependence of the EDC at the $\bar{\Gamma }$ point with the peak positions for samples 1 (**f**) and 4 (**g**), respectively. **h** Temperature dependence of the gap size of the DC for samples 1 and 4. The background color gradient shows the blunt transition from a massive to a massless DC and the error bars represent the uncertainty in the peak fitting.

  Figure 1a shows the typical band dispersion near the Fermi level (*E*_F_) of the Bi_2_Te_3_ film. The surface DC, as well as the bulk conduction band, is observed. When the heterostructure is formed after Mn and Te deposition, the features change drastically as can be seen in Fig. 1b. The original DC, which was buried in the bulk valence band, is pulled out into the bulk band gap. Furthermore, the DC now becomes massive with a gap of  ~75 meV, as can be noticed from the energy distribution curve (EDC) at the $\bar{\Gamma }$ point and the second derivative image in Fig. 1c. One can also notice that the DC dispersion becomes flat near *E*_F_ at *k* = 0.1 – 0.2 Å^−1^ in Fig. 1c. We have found that the massive DC is observed for all the samples we have fabricated, although the gap size Δ may be different; while sample 1 shown in Fig. 1b and c shows Δ ~75 meV, sample 2 shows Δ ~40 meV for *hν* = 8–21 eV (Fig. 1d and Supplementary Fig. 1), sample 3 shows Δ ~45 meV at *hν* = 15 eV (Supplementary Fig. 2a, d), and sample 4 shows Δ ~70 meV at *hν* = 9 eV (Supplementary Fig. 4a). Such scattering in the gap size has also been observed in a similar magnetic topological heterostructure of MnBi_2_Se_4_/Bi_2_Se_3_^10^ and likely results from the slight difference in the actual sample structure, which will be discussed later. Interestingly, at low photon energy, there is some photoemission intensity slightly outside of the massive DC as shown by the arrow in Fig. 1d. The details of the photon energy dependence can be found in Supplementary Fig. 1 and the reason of the appearance of this intensity will be discussed later too. We have performed spin- and angle-resolved photoemission spectroscopy (SARPES) measurements for sample 3, which is shown in Supplementary Fig. 2. It is obvious that the DC is spin-polarized and the spin-orientation is perpendicular to the wave number. Thus, we have found that the DC shape changes significantly by the deposition of Mn and Te on Bi_2_Te_3_. Namely, it becomes massive and the dispersion changes and becomes flatter near *E*_F_. It should also be emphasized that no additional trivial electronic states emerge in our novel heterostructure in contrast to the case of systems with sharp interfaces between a magnetic and topological insulator such as MnSe/Bi_2_Se_3_^25^, which have been intensively studied during the last several years.

To investigate the origin of the DC gap formation, we have performed temperature-dependent angle-resolved photoemission spectroscopy (ARPES) measurements. Figure 1e shows the DC dispersion of sample 1 measured at 290 K. The $\bar{\Gamma }$ point EDC shows that the DC is now massless as one intensity peak is revealed. This is in strong contrast to the MnBi_2_Se_4_/Bi_2_Se_3_ system, where the DC gap was clearly observed up to room temperature^10^. Figure 1f shows the temperature evolution of the close-up of the EDC near the DC gap or the Dirac point. Somehow, the midpoint of the DC gap is shifting away from the Fermi level for the spectra at lower temperature. Similar feature has been observed in the DC of the MnBi_2_Te_4_ (0001) surface system^13^. The peak positions for each spectrum have been determined by the analyses described in Supplementary Fig. 3. We can say that two peaks are observed up to 200 K, but most likely, only one can be identified for the spectra at 250 K and 290 K. We have performed further detailed temperature-dependent measurements, especially around 200 K, for sample 4 at *hν* = 9 eV as shown in Fig. 1g and Supplementary Fig. 4. As the sample temperature is increased, the spectral weight of the two peaks decreases accompanying the shrinking of the gap size to  ~40 meV. But at  ~210 K, the peak at higher binding energy starts to become sharp again and we think it most likely is a single component for the data at higher temperatures, although weak features can still be observed at 0.15 eV. This remanent hump may be due to (i) the coexistence of areas within the sample where the gap has closed and where it is still open (since this sample is actually a mixture of two phases) or/and (ii) the intermixing of Mn and Bi as described below. The deduced gap size for both samples is shown in Fig. 1h. The tendency of the gap shrinking, diminishing of the two clear peak structures at 100–200 K, and the peak resharpening above 200 K which ultimately seems to become a single peak at  ~250 K is a common characteristic for both samples 1 and 4. Thus we conclude that there is some kind of blunt phase transition at 200–250 K and the massive DC gradually becomes massless, with the gap ultimately disappearing at room temperature.

### Identification of the massive Dirac cone

**Fig. 2:**
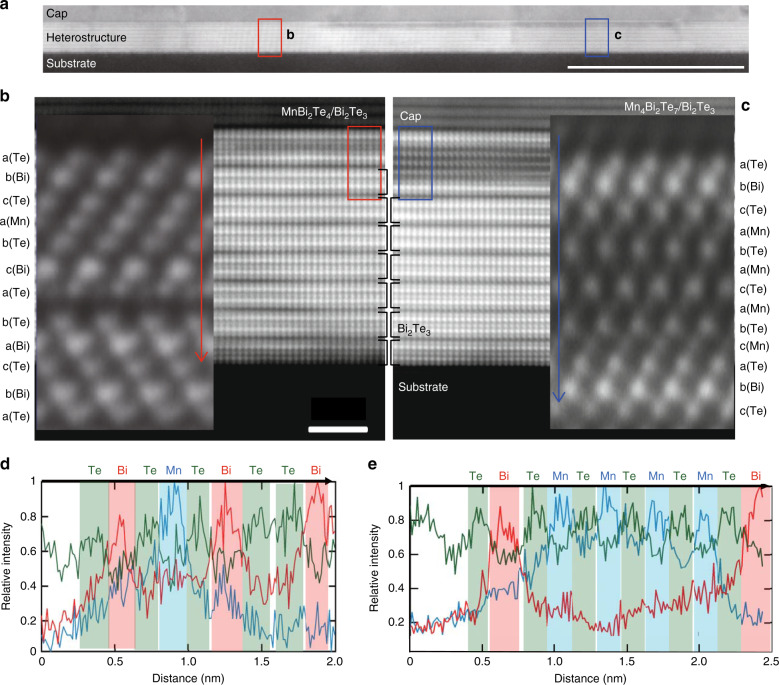
Atomic structure of the novel heterostructure. **a** Large scale HAADF-STEM image of the heterostructure measured at room temperature. The electron beam was incident along the [210] direction. Scale bar, 50 nm (horizontal white solid line). **b**, **c** Close-up image of region b and c in **a**, respectively. The inset shows the atomically-resolved image along the [110] direction showing the stacking sequence. The structure of the topmost layer is MnBi_2_Te_4_ (**b**) and Mn_4_Bi_2_Te_7_ (**c**), respectively. Scale bar, 2 nm (horizontal white solid line). **d**, **e** EDS mapping along the arrow in **b** (**d**) and **c** (**e**), respectively, showing the chemical composition of the heterostructure.

Otrokov et al., theoretically proposed that a massive DC with a gap of 77 meV will form for the MnBi_2_Te_4_/Bi_2_Te_3_ heterostructure^8^. When one compares the dispersion shown in Fig. 1c with this calculation, the experimental result is not consistent with the theoretical prediction since the lower DC of the calculation shows quite a flat dispersion while the experimental data shows a sharp dispersion similar to the upper DC. In order to verify the atomic structure of the present heterostructure, we have performed scanning transmission electron microscopy (STEM) observations. Figure 2a shows a low magnification STEM image of the sample showing the substrate Si, the heterostructure, and the capping layer. The incident electron beam was along the [210] direction. Upon close inspection, there seems to be two distinct areas distributed equally in the heterostructure; namely the region where the cap/heterostructure interface images brightly and the other region where it images darkly. The enlarged images from these two regions are shown in Fig. 2b and c, respectively. There are quintuple atomic layers stacking in the Bi_2_Te_3_ layer. However, the topmost layer seems to be composed of seven atomic layers in b, whereas it seems to be a 13 layer block in c.

To gain further insight, we have performed high-resolution high-angle annular dark field STEM (HAADF-STEM) observation from the [110] direction as shown in the insets of Fig. 2b, c. Energy-dispersive X-ray spectroscopy (EDS) measurements were also performed to verify the atomic composition as shown in Fig. 2d and e for the structures of Fig. 2b, c, respectively. As a result it has been revealed that Fig. 2b is the MnBi_2_Te_4_/[7QL Bi_2_Te_3_] structure with the abcabca-bacba stacking, while Fig. 2c shows the Mn_4_Bi_2_Te_7_/[6QL Bi_2_Te_3_] with the abcabacabcabc stacking. To say it simply, the septuple layer block has only one Mn–Te bilayer whereas the 13 layer block is a structure with four Mn–Te layers incorporated into Bi_2_Te_3_. The NaCl-type hexagonal stacking (abcabcabc...) is somehow disturbed in the former heterostructure whereas the latter is a hybrid of the NaCl (top three Te–Bi–Te layers) and NiAs-type stacking (abacabac..., Mn–Te). In ref. ^24^, it was reported that the NiAs-type stacking is energetically favored compared to the NaCl-type in the Mn–Te multilayers and is consistent with the experimental observation. The consequence of the realization of this interesting stacking for the magnetic order of the heterostructure will be discussed later.

We should mention that there were some regions which showed the incorporation of two or three Mn–Te bilayers in the topmost Bi_2_Te_3_ QL, but the possibility to find them was very small. We think that when the Mn and Te deposition exceeds the mono-bilayer, the quadruple-bilayer will be formed since the system tends to maintain similar height as the sum of the topmost septuple and the QL so that the two structures can connect with a smooth interface. Such a growth allows minimizing the surface energy owing to the disappearance of the steps initially presented on Bi_2_Te_3_ surface as schematically shown in Supplementary Fig. 5. We believe that the mixing between Bi and Mn is also unavoidable, especially at the topmost Bi layers (Fig. 2d and e). Such intermixing between Bi and Mn has also been reported for bulk MnBi_2_Te_4_^12,13^. This may be one of the reasons for the scattering of the measured DC gap size in ARPES.

**Fig. 3:**
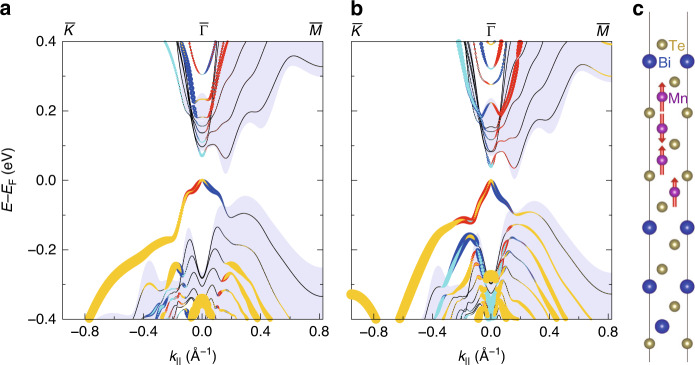
Ab initio calculations. **a** Calculated band dispersion of the MnBi_2_Te_4_/Bi_2_Te_3_ heterostructure shown in Fig. 2. The Mn layer was assumed to be out-of-plane ferromagnetic. **b** Calculated band dispersion of the Mn_4_Bi_2_Te_7_/Bi_2_Te_3_ heterostructure shown in Fig. 2. The Mn layers were assumed to have the spin-orientation as shown in (**c**). **c** Spin configuration of the Mn_4_Bi_2_Te_7_/Bi_2_Te_3_, which has the lowest energy. The adjacent Mn monolayers are coupled both ferromagnetically and antiferromagnetically.

Now that the atomic structure has been resolved, the band dispersion for the two structures in Fig. 2 was calculated as shown in Fig. 3a and b, respectively. In both cases, the out-of-plane magnetization was assumed based on the experimental data discussed later. For the Mn_4_Bi_2_Te_7_/Bi_2_Te_3_ shown in Fig. 3b, a complicated magnetic configuration was assumed with both ferromagnetic (FM) and antiferromagnetic (AFM) couplings between adjacent layers as shown in Fig. 3c. We found this magnetic structure to be energetically most favorable compared to those with only FM or only AFM coupled spin structures and likely originates from the interesting atomic stacking observed in Fig. 2c. The overall shape is similar between Fig. 3a, b but Δ is  ~70 meV in a whereas it is about 40 meV in b. Since the spot size in ARPES was larger than the domain size of the two heterostructures, we indeed observed DCs from both for a particular measurement condition as is shown in Fig. 1d and Supplementary Fig. 4. To identify the origin of the observed DCs, we have performed ARPES measurements for samples with different amounts of Mn and Te deposition as shown in Supplementary Figs. 6 and 7. The DC dispersion of the pure MnBi_2_Te_4_/Bi_2_Te_3_ phase is not so different from the Bi_2_Te_3_ substrate and there is no gap opening at 16 K for this sample. This dispersion is quite similar to the additional feature observed outside of the massive DC in Fig. 1d and Supplementary Fig. 1 at low photon energy and is likely to correspond to the DC of MnBi_2_Te_4_/Bi_2_Te_3_. Since in ref. ^12^, the DC of the heterostructure consisting of MnBi_2_Te_4_ and Bi_2_Te_3_ showed a gap closing at 20 K with a Curie temperature of 10 K, it is most likely that the DC of our MnBi_2_Te_4_/Bi_2_Te_3_ heterostructure remains massless because the Curie temperature is not reached at 16 K. As such, we can say that the massive DC extensively studied in Fig. 1 originates from the Mn_4_Bi_2_Te_7_/Bi_2_Te_3_ heterostructure.

### Magnetism and its relation with the Dirac-cone gap

**Fig. 4:**
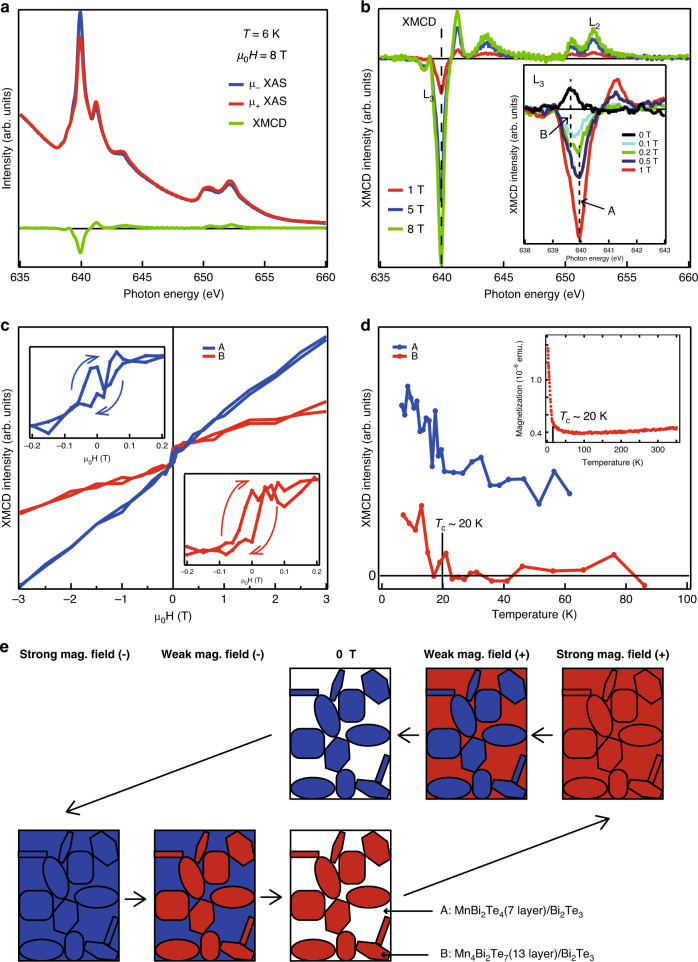
Magnetic property of the novel heterostructure. **a** X-ray absorption spectra (XAS) measured at 6 K for a circularly polarized incident light when a  ±8 T magnetic field was applied along the sample surface-normal direction. *μ*_+_ and *μ*_−_ correspond to the spectrum obtained with left and right-handed circularly polarized photons, respectively. The corresponding XMCD spectra is also shown. The Te capping layer was removed by annealing the sample in UHV. **b** Magnetic field dependence of the XMCD spectra. The inset shows the enlarged spectra for small magnetic fields. The peak position is clearly different between high (A) and low (B) fields and coexists in the spectra at 0.2 T, indicating that there are two distinct Mn components. At remnant magnetization (0 T), the XMCD signal changes signs. **c** Magnetic field dependence of the XMCD signal of the A and B components measured at 6 K. B shows a clear negative hysteresis. **d** Temperature dependence of the XMCD signal of the A and B components measured at 0.2 T. The Curie temperature of B is determined as  ~20 K, while A shows a paramagnetic dependence and a finite XMCD signal is observed even at 60 K. The inset shows the temperature dependence of the magnetization measured with SQUID. **e** Schematic drawing of the field-dependent magnetic state of the present system. The antiferromagnetic coupling between the two heterostructures that manifests itself at low field is likely responsible for the DC gap opening/closing.

Finally, we have performed magnetic measurements to verify the relationship between the Dirac-cone gap opening/closing and the magnetic property of the system. Figure 4a shows the X-ray absorption spectroscopy (XAS) spectra taken at 6 K with a magnetic field of 8 T applied perpendicular to the sample at the Mn *L* edge. *μ*_+_ and *μ*_−_ correspond to the spectrum obtained with left and right-handed circularly polarized photons, respectively. The corresponding X-ray magnetic circular dichroism (XMCD) spectrum is also shown together and a clear signal is detected. Figure 4b shows the magnetic field dependence of the XMCD spectra. The peak signal decreases for smaller fields. The inset shows the evolution of the *L*_3_ peak for the fields smaller than 1 T. One can notice that at 0 T, the sign of the *L*_3_ peak reverses and becomes positive. This means that the remanent magnetization is opposite to the applied field (negative remanence). Moreover, we can find that the peak position changed by decreasing the field. Namely, the peak position was 639.9 eV for the fields larger than 0.5 T (peak A), but at 0.2 T a shoulder structure can be found at 639.5 eV which becomes dominant at 0.1 T and 0 T (peak B). Thus this clearly reveals that there are two different Mn components involved in the magnetization of this system.

To gain further insight into the intricate magnetism of this system, we have performed detailed field dependent XMCD measurements for peaks A and B as shown in Fig. 4c (*M*–*H* curves). In order to display the magnetization change near zero-field clearly, we show the close-up for −0.2 to 0.2 T. Peak B shows a clear hysteresis although it is clockwise reflecting the negative remanence which is called an inverted hysteresis or proteresis^26^. For peak A, the signal intensity is nearly proportional to the field strength although it also shows some anomaly near zero-field albeit not a clear hysteresis. While it is difficult to make a definite conclusion, this is most likely to be caused by the finite peak width of peak B. Figure 4d shows the temperature dependence of the XMCD signal for peaks A and B measured at 0.2 T (*M*–*T* curves). For peak B, the signal drops to nearly zero at  ~20 K. On the other hand, the XMCD intensity of peak A decreases for higher temperature but is still finite up to 60 K. Taking into account all these facts, peak A corresponds to the Mn component that is paramagnetic down to 6 K while peak B seem to be reflecting the ferromagnetic Mn components of the system.

Let us now discuss the magnetic properties obtained from XMCD measurements by comparing with the atomic structure. As we have clarified, there are two regions with different atomic structures inside the sample: MnBi_2_Te_4_ and Mn_4_Bi_2_Te_7_ blocks. It is natural to interpret that peaks A and B correspond to the Mn components in these two different heterostructures. Since the clear DC gap was only observed for Mn_4_Bi_2_Te_7_ (Figs. 1, S1, S2) but not for the MnBi_2_Te_4_(Supplementary Fig. 6) down to 16 K, we speculate that the time-reversal symmetry broken ferromagnetic component (peak B) corresponds to the Mn of Mn_4_Bi_2_Te_7_. (We will call the Mn components that correspond to these two peaks as Mn A and B from now on.) However, the experimentally determined Curie temperature of  ~20 K for Mn B is much lower than the temperature that the DC gap gradually closes and seems to become massless (200–250 K). In fact, we have performed temperature-dependent SQUID measurements from 4 K to 350 K and found that there is no anomaly in the sample magnetization above 20 K, which is consistent with the XMCD data (inset of Fig. 4d). Even at 30 K, which is clearly above the observed Curie temperature, the ARPES spectra show prominent peaks as shown in Fig. 1f and g. These facts suggest that the loss of the long-range magnetic order and the DC gap closing in this system is not correlated with each other.

The origin of the inverted hysteresis and the negative remanence below *T*_c_ may be explained by the antiferromagnetic coupling between domains of Mn A and B as shown in Fig. 4e. At high magnetic field, both align parallel to the field direction although maybe not completely spin-flopped^27^. However, when the field is decreased, Mn A components may still be aligned parallel to the field but Mn B can align in the opposite direction since they feel the magnetic moment of the surrounding Mn A components stronger than the applied field and couple antiferromagnetically to them. This kind of explanation has been employed for various systems showing proteresis such as Co nanoparticles^26,28^ and may also be applied to the present system, although the size of the magnetic domains in the present sample is at least ten times larger than that of the nanoparticles. The persistence of this antiferromagnetic interaction may be one possible origin of the gap opening above *T*_c_. Another possibility is that the magnetic domains are fluctuating at temperatures higher than *T*_c_ similar to the case of bulk MnBi_2_Te_4_^13,16,22^. While further study is needed to clarify this issue, we would like to emphasize that our study clearly shows that the magnetic DC gap does eventually close even though it occurs at a much higher than the magnetic critical temperature.

In conclusion, we fabricated a novel magnetic topological heterostructures by depositing Mn and Te on Bi_2_Te_3_. The fabricated samples were a mixture of MnBi_2_Te_4_/Bi_2_Te_3_ and Mn_4_Bi_2_Te_7_/Bi_2_Te_3_. We found that the surface DC of the latter was massive with a gap of 40–75–meV at 16 K and decreased for higher temperature. A blunt transition to a massless DC was observed at 200–250 K. However, the Curie temperature of the system was much lower (~20 K). Thus, our results show for the first time that the Dirac point gap of the interface topological state of a magnetic topological heterostructure eventually closes, albeit at a temperature well above the magnetic *T*_c_.

## Methods

### Sample fabrication

The heterostructure samples were prepared by molecular beam epitaxy in ultrahigh vacuum (UHV) chambers equipped with a reflection-high-energy electron diffraction (RHEED) system. First, a clean Si(111)-7 × 7 surface was prepared on an *n*-type substrate by a cycle of resistive heat treatments. Then Bi was deposited on the 7 × 7 substrate at  ~250 °C in a Te-rich condition. Such a procedure is reported to result in a smooth epitaxial film formation with the stoichiometric ratio of Bi:Te = 2:3. The grown Bi_2_Te_3_ films were annealed at  ~250 °C for 5 min. The thickness of the Bi_2_Te_3_ films in this work is  ~8 QL. Finally, Mn was deposited on Bi_2_Te_3_ in a Te-rich condition at  ~260 °C. The 1 × 1 periodicity with the same lattice constant is maintained during this process for the samples we have fabricated.

For the X-ray magnetic circular dichroism (XMCD), superconducting quantum interference device (SQUID) and scanning transmission emission microscopy (STEM) measurements, the fabricated samples were first characterized with ARPES at room temperature. After confirming that the desired band dispersion is obtained (Fig. 1e and Supplementary Fig. 7), they were capped with ~10 nm of Te before taking them out of the UHV chamber.

### High-resolution and spin-resolved ARPES

Angle resolved photoemission spectroscopy (ARPES) and spin-resolved ARPES (SARPES) measurements were performed in situ after the sample preparation. The ARPES measurements were performed at BL-7U of UVSOR-III using *p*-polarized photons in the energy range of 7.5–21 eV^29^, as well as at BL-9B of HiSOR in the energy range of 15–21 eV. All the data shown were taken at 16 K unless otherwise indicated. The energy and angular resolutions were 15 meV and 0.15°, respectively. The SARPES measurements were performed at BL-9B of HiSOR and the energy and angular resolutions were 30 meV and 0.75°, respectively^30^.

### Magnetic characterization

The SQUID measurements were conducted with a commercial MPMS-52 system (Quantum Design).

The X-ray absorption spectroscopy (XAS) and XMCD measurements were performed at BL-23SU of SPring-8^31^ and BL-16A of KEK-PF^32^ with circularly polarized X-ray radiation. The total-electron yield mode was employed in both cases. The Te-capped samples were annealed at ~250 °C to remove the capping layers prior to the measurements.

### Structure analysis

Electron transparent specimens for STEM observations were prepared by the standard lift-out technique using an FEI Helios G4-UX dual-beam system. Probe abberation corrected STEM, FEI TitanG2 80–200 microscope, was used. Chemical compositions were measured by energy-dispersive X-ray spectroscopy (EDS).

### Theoretical calculation

Electronic structure calculations were carried out within the density functional theory using the projector augmented-wave (PAW) method^33^ as implemented in the VASP code^34–36^. The exchange-correlation energy was treated using the generalized gradient approximation^37^. The Hamiltonian contained scalar relativistic corrections and the SOC was taken into account by the second variation method^38^. In order to describe the van der Waals interactions, we made use of the DFT-D3^39,40^ approach. The energy cutoff for the plane-wave expansion was set to 270 eV. All structural optimizations were performed using a conjugate-gradient algorithm and a force tolerance criterion for convergence of 0.01 eV/Å. SOC was always included when performing relaxations. The Mn 3*d*-states were treated employing the GGA+*U* approach^41^ within the Dudarev scheme^42^. The *U*_eff_ = *U* − *J* value for the Mn 3*d*-states was chosen to be equal to 5.34 eV, as in previous works on similar systems^8,13,14,24^.

## Supplementary information

Supplementary Information

## Data Availability

The datasets generated during and/or analyzed during the current study are available from the corresponding author on reasonable request.
