# Supplementary material for: Fabrication of a novel magnetic topological heterostructure and temperature evolution of its massive Dirac cone
Source: Nat Commun. 2020 Sep 24;11:4821. doi: 10.1038/s41467-020-18645-9 (PMC7515900; doi:10.1038/s41467-020-18645-9)
Supplement: Supplementary file 1 — Supplementary Information [file 41467_2020_18645_MOESM1_ESM.pdf]

Supplementary Information

Fabrication of a novel magnetic topological heterostructure and temperature evolution of its massive Dirac cone

T. Hirahara et al.,

Supplementary Note 1: Photon energy dependence

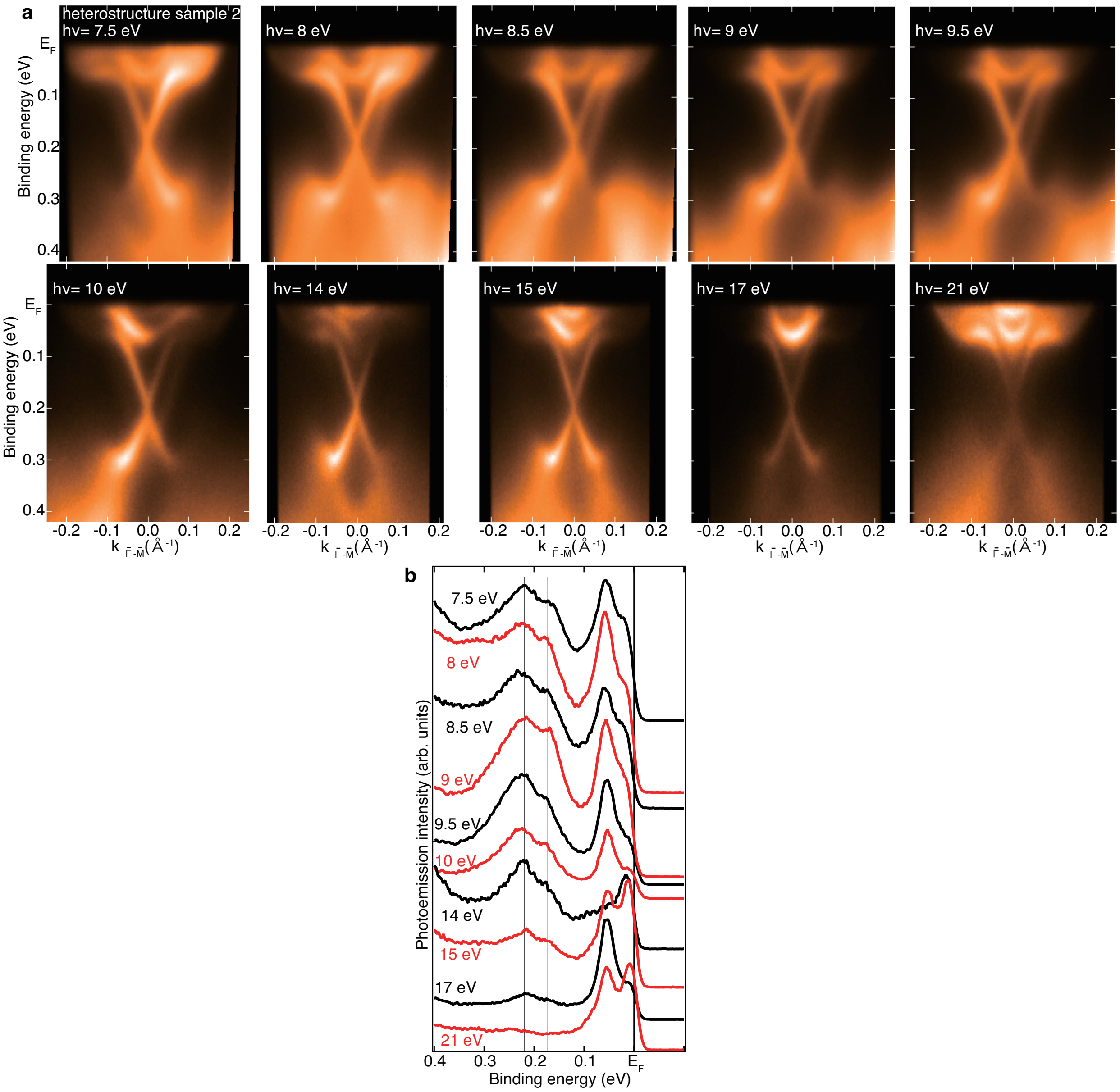

Supplementary Figure 1: **Photon energy dependence of ARPES image of the Dirac cone.** (a) Band dispersion image measured for heterostructure sample 2 at $h\nu$ = 7.5, 8, 8.5, 9, 9.5, 10, 14, 15, 17, and 21 eV. All the measurements were performed at 30 K. (b) EDC curves at the $\bar{\Gamma}$ point of the images shown in (a).

Supplementary Figure 1a shows the band dispersion image of the Mn, Te/Bi_2Te_3 heterostructure sample 2 taken with different photon energies ($h\nu$ =7.5, 8, 8.5, 9, 9.5, 10, 14, 15, 17, and 21 eV). The Dirac cone is $h\nu$ independent and can be safely regarded as surface

states. Supplementary Figure 1b shows the EDC spectra at the $\bar{\Gamma}$ point of the images shown in a and one can notice that the two peaks of the massive Dirac cone can be recognized for all the photon energies as indicated by the black lines, although there is variation in the peak intensity. At low energy, additional features outside the gapped Dirac cone appear. The features near E_F near the $\bar{\Gamma}$ point become strong at 21 eV and it is extremely difficult to see the gap in the EDC curve at the $\bar{\Gamma}$ point.

Supplementary Note 2: Spin-resolved ARPES

Supplementary Figure 2 shows the spin-resolved ARPES results for the Mn, Te/Bi_2Te_3 heterostructure sample 3. The gap size in this sample is $\sim$ 40 meV (see the EDC in Supplementary Fig. 2a. The measurements were performed along the $\bar{\Gamma} - \bar{\mathrm{M}}$ (x in Supplementary Fig. 2b) direction and the spin-orientation along the y direction was measured as shown in Supplementary Fig. 2c for $\theta = -4$ to $+4°$. Supplementary Figure 2 d shows the results obtained in c overlapped on the band dispersion image. Red and blue markers represent the spin-up and down states that are antisymmetric with respect to the $\bar{\Gamma}$ point, respectively. On the other hand, brown and purple markers represent spin-up and down states that are not antisymmetric and likely show spin-polarization due to spin-dependent photoemission dipole matrix element effect [1, 2]. The Dirac cone shows the expected antisymmetric spin-split structure.

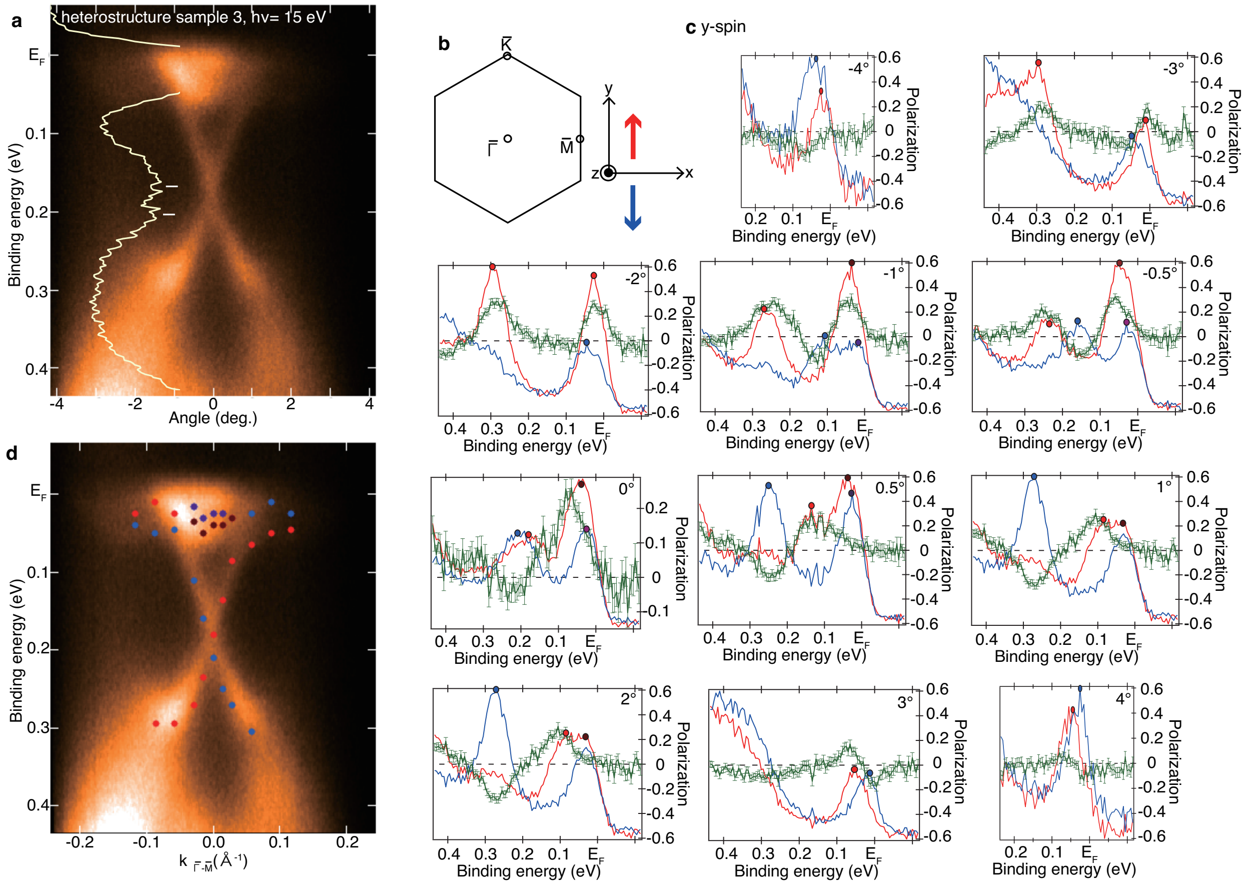

Supplementary Figure 2: **Spin-resolved ARPES spectra.** (a) Band dispersion image ($E - \theta$) of heterostructure sample 3. (b) The Brillouin zone shown together with the definition of the coordinates. (c) SARPES spectra and spin-polarization results for $\theta = -4$ to $+4°$. (d) The spin-split band dispersion of sample 3 obtained from SARPES (c) overlaid on the $E - k$ band dispersion image. Red and blue markers represent the spin-up and down states that are asymmetric with respect to the $\bar{\Gamma}$ point, respectively. Brown and purple markers represent spin-up and down states that do not have an asymmetric spin structure around $\bar{\Gamma}$. All the measurements were performed at 30 K.

Supplementary Note 3: Quantitative analyses for peak determination to deduce the Dirac-cone gap size

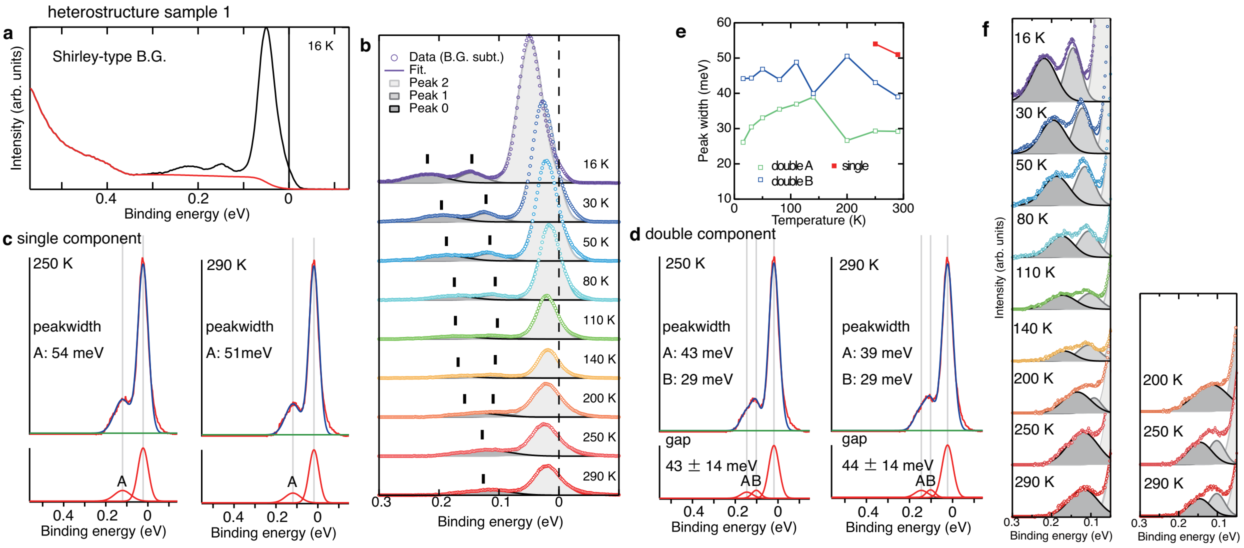

Supplementary Figure 3: Quantitative analysis of the Dirac cone. (a) The $\bar{\Gamma}$ point EDC at 16 K for sample 1. A Shirley-type background is drawn together. (b) Results of the peak fitting after the background was subtracted from the raw data. Gaussian functions were used. (c) Close-up of the fitted spectra at 250 and 290 K shown in b. Double components were assumed near the Dirac cone crossing. (d) Close-up of the fitted spectra at 250 and 290 K when a single component was assumed near the Dirac cone crossing. (e) The temperature dependence of the fitted peak width. (f) Zoom in of the spectral features shown in b-d for better visualization of the fitting results at the energy range of 0.1-0.3 eV. The left panel shows the data shown in b and the right panel shows the fitted results when the number of peaks at 0.1-0.3 eV used in the fitting have been changed. Namely, we used two, one, and one peak(s) in the fitting for the data at 200 K, 250 K, and 290 K in the left panel respectively, while it was changed to one, two and two in the right panel.

Supplementary Figure 3 shows the details of the quantitative analyses for peak determination to deduce the Dirac-cone gap size. First, a Shirley-type background was estimated from the raw spectra as shown in Supplementary Fig. 3a for the data taken at 16 K. After subtracting the background, the spectra were fitted multiple Gaussian peaks (Supplementary Fig. 3b). We first fitted the experimental data with 3 peaks (including the

strong peak α just below E_F) for all the spectra (Supplementary Fig. 3c). By deducing the peak width of the fitted results, we found a jump between 140 and 200 K. The width of peak α increased significantly, whereas that for peak B decreased although it was monotonously increasing below 140 K (Supplementary Fig. 3e). Since the spectra features of the Dirac cone shown in Fig. 1f for temperatures higher than 200 K could also be assumed as a single peak, we also tried fitting using only 2 components, as shown in Supplementary Fig. 3d. In this case, the peak width was larger than in the case when 3 components were used. Especially the data at 200 K showed a single peak width of 64 meV, much larger than the other fitted results. On the other hand, for the data of 250 and 290 K, the results were fairly reasonable considering the temperature broadening. Since the temperature broadening effect alone cannot explain the fact that the peak width for the spectrum at 200 K is larger than those for higher temperature, we believe that a gradual phase transition is occurring at 200-250 K. The raw data in Fig. 1f shows that the peak becomes sharper at 250 and 290 K than those at 200 and 140 K. Such feature is also observed in sample 4 (Fig. 1g), reinforcing our statement. Taking all these facts into account, we assigned 3 peaks to the spectra below 200 K and deduced the gap size from the analysis, whereas only 2 peaks were considered for the spectra at 250 and 290 K, meaning the closing of the Dirac cone gap. Although the transition at 200-250 K is dull, we believe that the gap of the massive Dirac cone at low temperature has closed at 290 K. Supplementary Figure 3f shows the zoom in of the fitted results shown in b-d for better visualization of the fitting results at the energy range of 0.1-0.3 eV. The left panel shows the data shown in b and the right panel shows the fitted results when the number of peaks at 0.1-0.3 eV used in the fitting have been changed. Namely, we used two, one, and one peak(s) in the fitting for the data at 200 K, 250 K, and 290 K in the left panel respectively, while it was changed to one, two and two in the right panel.

Supplementary Note 4: Band dispersion image for heterostructure sample 4

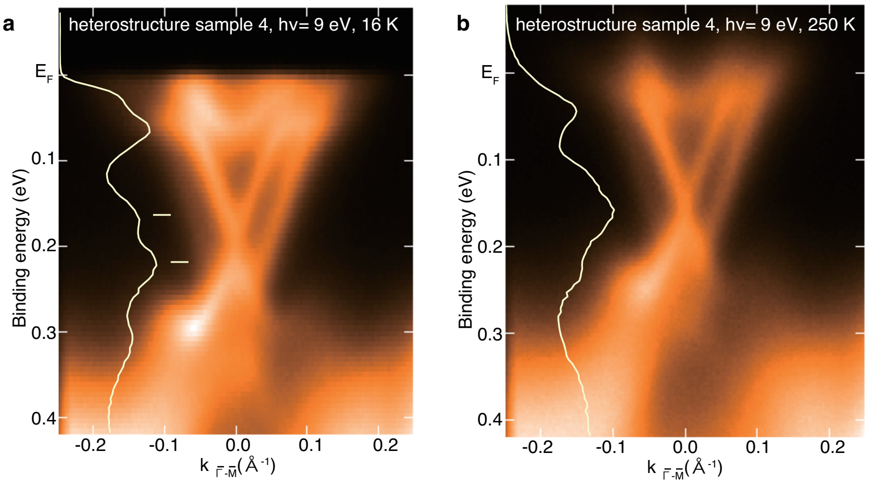

Supplementary Figure 4: Temperature dependence of the ARPES image for heterostructure sample 4. Band dispersion image of sample 4 measured with $h\nu = 9$ eV photons at 16 K (a) and at 250 K (b), respectively. The solid lines show the EDC at the $\bar{\Gamma}$ point, representing that the Dirac cone is massive with a 70 meV gap in (a). There is additional feature outside the clearly observed DC which corresponds to the DC of the $MnBi_2Te_4/Bi_2Te_3$ heterostructure as discussed in the main text.

Supplementary Figures 4a and b show the band dispersion image of sample 4 measured with $h\nu = 9$ eV photons at 16 K (a) and at 250 K (b), respectively. Due to the low photon energy, the feature outside the Dirac cone can be clearly seen on the positive side. The solid lines show the EDC at the $\bar{\Gamma}$ point. In Supplementary Fig. 4a, the Dirac cone is massive with a 70 meV gap. The whole data set of the temperature dependence of the EDC can be found in Fig. 1g.

Supplementary Note 5: Possible origin of the formation of the 13 layer block

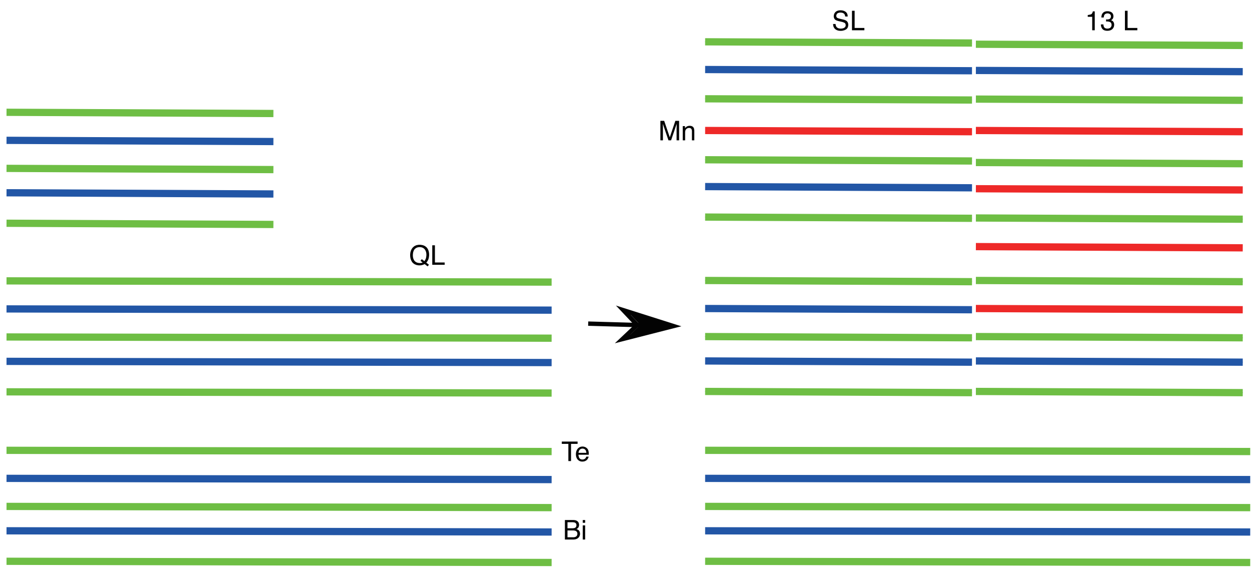

Supplementary Figure 5: **Schematic drawing concerning the Mn and Te incorporation into Bi_2Te_3.**

Supplementary Figure 5 shows a schematic drawing depicting the possible way of how Mn and Te layers can be incorporated into Bi_2Te_3. Most likely, the Mn and Te layers are first inserted into the middle of the septuple layer. Then with further deposition, they presumably stack to minimize the steps that are initially present on the pristine Bi_2Te_3 surface. As a result, the 13 layer block with 4 Mn-Te layers stacked is formed since the height of the 13 layer block is nearly equal to that of the sum of the quintuple and the septuple layers.

Supplementary Note 6: Band dispersion image of the $MnBi_2Te_4/Bi_2Te_3$ heterostructure

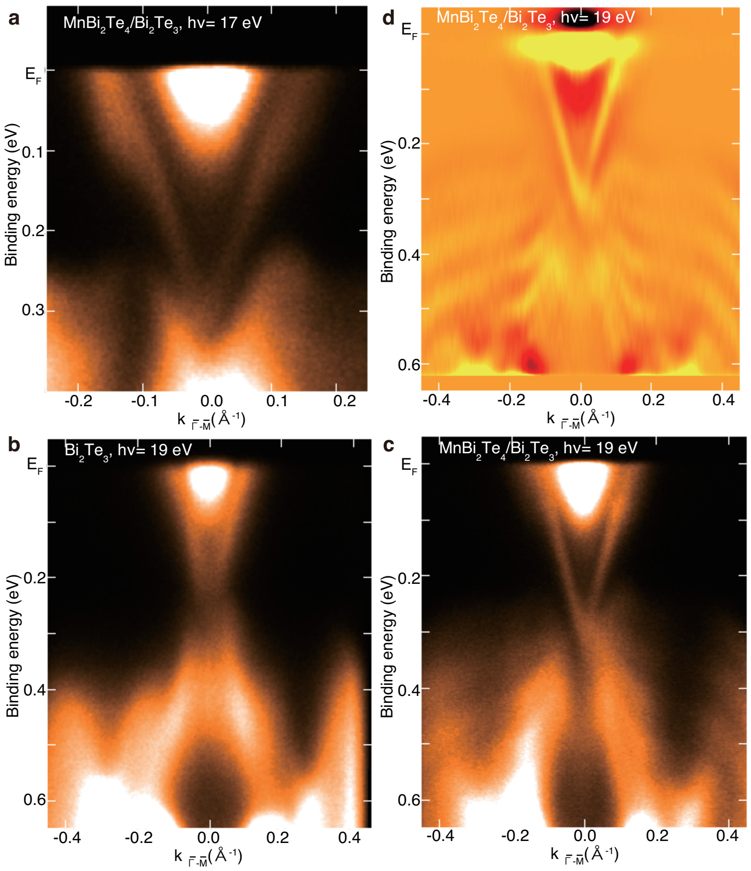

Supplementary Figure 6: **ARPES image of $MnBi_2Te_4/Bi_2Te_3$.** (a) Band dispersion image of the $MnBi_2Te_4/Bi_2Te_3$ heterostructure measured at $h\nu$=17 eV. (b, c) Comparison of the band dispersion of Bi_2Te_3 (b) and $MnBi_2Te_4/Bi_2Te_3$ (c) in a larger region of the $E-k$ space. (d) The second derivative with respect to the energy of the band dispersion image in (c). All the measurements were performed at 16 K.

Supplementary Figure 6 shows the band dispersion image for the $MnBi_2Te_4/Bi_2Te_3$ heterostrcture. The sample was fabricated by decreasing the deposition time of Mn and Te and the septuple layer structure was confirmed with LEED I-V analyses (unpublished). It is quite similar to the substrate Bi_2Te_3 shown in Fig. 1a. The only difference is that the Dirac point has shifted slightly to higher binding energy. To emphasize the difference between Bi_2Te_3 and $MnBi_2Te_4/Bi_2Te_3$, we show in Figs. 6b and c the respective band dispersion

image in a larger region of the $E-k$ space. Supplementary Figure 6d is the second derivative with respect to the energy of the band dispersion image in c. The Dirac point shift can be recognized as well as the appearance of "skeleton" like features at finite wavenumber below 0.3 eV, which was absent for Bi_2Te_3.

The band dispersion image in Figs. 6a and c does not show a clear gap opening in contrast to the theoretical prediction in Ref. [3]. As discussed in the main text, this may be due to the fact that the measurement temperature of 16 K is above the Curie temperature of the system as reported in Ref. [4]. In fact, Ref. [5] predicts that the Curie temperature of $MnBi_2Te_4$ is 12 K from Monte Carlo simulations. For bulk $MnBi_2Te_4$, the Neel temperature is reported as 25 K [6]. However, this system is paramagnetic down to 6 K as shown in Fig. 4 and the origin of this low critical temperature compared to systems with similar crystal structure and the calculation should be an interesting research topic in the future.

Supplementary Note 7: Thickness dependence of the band dispersion of the Mn, Te deposited Bi_2Te_3 samples having different ratio of $MnBi_2Te_4$ and $Mn_4Bi_2Te_7$

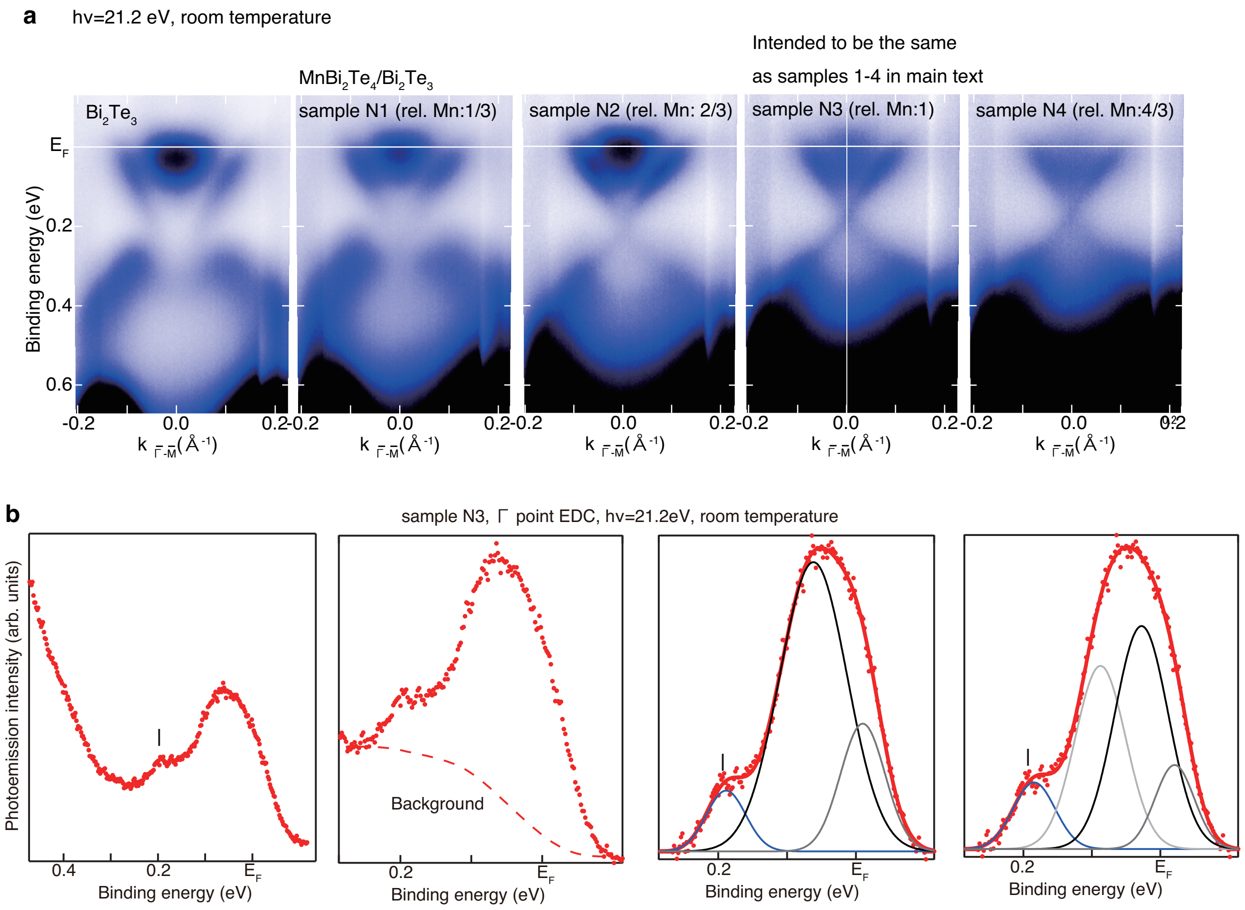

Supplementary Figure 7: Thickness dependence of the Mn, Te deposited Bi_2Te_3 samples. (a) Band dispersion of the Mn, Te deposited Bi_2Te_3 samples having different ratios of $MnBi_2Te_4$ and $Mn_4Bi_2Te_7$. (b) EDC curve at the $\bar{\Gamma}$ point for sample N3 and the analysis of peak fitting.

Supplementary Figure 7a shows the band dispersion of the Mn, Te deposited Bi_2Te_3 samples having different ratios of $MnBi_2Te_4$ and $Mn_4Bi_2Te_7$. The measurements were performed in the lab with HeI photons (21.2 eV) at room temperature. Starting from the substrate Bi_2Te_3 film, the relative Mn amount increases as 1/3, 2/3, 1, 4/3 from sample N1 to N4. Sample N3 was the one we intended to make repeatedly and is nearly the same as samples 1-4 shown in Figs. 1 and Supplementary Figs. 1, 2, 3, 4 (ratio of $MnBi_2Te_4$: $Mn_4Bi_2Te_7$ is 1:1). Sample N1 was determined as the single $MnBi_2Te_4$ phase from LEED

I-V measurements and the dispersion measured for the sample made in a similar manner at 16 K is shown in Supplementary Fig. 5. We also tried to perform LEED I-V analysis for sample N4 which we anticipated to be the single $Mn_4Bi_2Te_7$ phase, but we could not obtain good experimental data. Since the signal-to-noise ratio in the ARPES images also become low for sample N4 in Supplementary Fig. 7, it seems likely that to obtain a high-quality $Mn_4Bi_2Te_7$ sample is not possible for some reason and the quality of the experimental data for samples with larger amounts of $Mn_4Bi_2Te_7$ is also not high enough to make a detailed discussion on the DC gap.

Supplementary Figure 7b shows the EDC curve at the $\bar{\Gamma}$ point for sample N3 and the analysis of peak fitting. There is clearly only one peak for the DC whereas the features near E_F (bulk) can be fitted by either two or three components. This clearly means that the DC gap is absent at room temperature.

---

[1] Jozwiak, C., et al. Photoelectron spin-flipping and texture manipulation in a topological insulator. *Nature Physics* **9**, 293 (2013).

[2] Jozwiak, C., et al. Widespread spin polarization effects in photoemission from topological insulators. *Phys. Rev. B* **84**, 165113 (2011).

[3] Otrokov, M. M. et al. Highly-ordered wide bandgap materials for quantized anomalous Hall and magnetoelectric effects. *2D Mater.* **4** 025082 (2017).

[4] Rienks, E. D. L. et al. Large magnetic gap at the Dirac point in $Bi_2Te_3/MnBi_2Te_4$ heterostructures. *Nature* **576**, 423 (2019).

[5] Otrokov, M. M. et al. Unique Thickness-Dependent Properties of the van der Waals Interlayer Antiferromagnet $MnBi_2Te_4$ Films. *Phys. Rev. Lett.* **122**, 107202 (2019).

[6] Otrokov, M. M. et al. Prediction and observation of an antiferromagnetic topological insulator. *Nature* **576**, 416 (2019).
